# Advancing Toward P6 Medicine: Recommendations for Integrating Artificial Intelligence in Internal Medicine

**DOI:** 10.3390/clinpract15110200

**Published:** 2025-10-29

**Authors:** Ismael Said-Criado, Filomena Pietrantonio, Marco Montagna, Francesco Rosiello, Oleg Missikoff, Carlo Drago, Tiffany I. Leung, Antonio Vinci, Alessandro Signorini, Ricardo Gómez-Huelgas

**Affiliations:** 1 Palliative Care Unit, Internal Medicine Department, Pontevedra’s Hospital, Healthcare Research Institute Galicia Sur, 36001 Pontevedra, Spain; ismael.said.criado@sergas.es; 2 Internal Medicine Unit, Medical Area Department, Castelli Hospital, ASL Roma 6, Ariccia, 00040 Rome, Italy; 3 Departmental Faculty of Medicine, Saint Camillus International University of Health Sciences (Unicamillus), 00153 Rome, Italy; 4 Department of Medicine, Vita-Salute San Raffaele University, 20132 Milan, Italy; montagna.marco@hsr.it; 5 Department of Public Health and Internal Medicine, Sapienza Università di Roma, 00185 Rome, Italy; francesco.rosiello@uniroma1.it; 6 Department of European, American and Intercultural Studies, Sapienza University of Rome, 00185 Rome, Italy; oleg.missikoff@uniroma1.it; 7 Department of Economics, Psychology, Communication, Education and Motor Sciences, Niccolò Cusano University, 00166 Rome, Italy; carlo.drago@unicusano.it; 8 Department of Internal Medicine (Adjunct), Southern Illinois University School of Medicine, Springfield, IL 62794, USA; 9 Doctoral School in Nursing Sciences and Public Health, University of Rome “Tor Vergata”, 00133 Rome, Italy; antonio.vinci.at@hotmail.it; 10 Health Technology Assessment (HTA) Unit, Saint Camillus International University of Health Sciences, 00131 Rome, Italy; alessandro.signorini@unicamillus.org; 11 Internal Medicine Department, Hospital Regional Universitario de Málaga, University of Málaga, 29010 Malaga, Spain; ricardogomezhuelgas@hotmail.com

**Keywords:** artificial intelligence, internal medicine, P6 Medicine, Delphi method, SWOT analysis

## Abstract

Background: Internists formulate diagnostic hypotheses and personalized treatment plans by integrating data from a comprehensive clinical interview, reviewing a patient’s medical history, physical examination and findings from complementary tests. The patient treatment life cycle generates a significant volume of data points that can offer valuable insights to improve patient care by guiding clinical decision-making. Artificial Intelligence (AI) and, in particular, Generative AI (GAI), are promising tools in this regard, particularly after the introduction of Large Language Models. The European Federation of Internal Medicine (EFIM) recognizes the transformative impact of AI in leveraging clinical data and advancing the field of internal medicine. This position paper from the EFIM explores how AI can be applied to achieve the goals of P6 Medicine principles in internal medicine. P6 Medicine is an advanced healthcare model that extends the concept of Personalized Medicine toward a holistic, predictive, patient-centered approach that also integrates psycho-cognitive and socially responsible dimensions. An additional concept introduced is that of Digital Therapies (DTx), software applications designed to prevent and manage diseases and disorders through AI, which are used in the clinical setting if validated by rigorous research studies. Methods: The literature examining the relationship between AI and Internal Medicine was investigated through a bibliometric analysis. The themes identified in the literature review were further examined through the Delphi method. Thirty international AI and Internal Medicine experts constituted the Delphi panel. Results: Delphi results were summarized in a SWOT Analysis. The evidence is that through extensive data analysis, diagnostic capacity, drug development and patient tracking are increased. Conclusions: The panel unanimously considered AI in Internal Medicine as an opportunity, achieving a complete consensus on the matter. AI-driven solutions, including clinical applications of GAI and DTx, hold the potential to strongly change internal medicine by streamlining workflows, enhancing patient care and generating valuable data.

## 1. Introduction

The practice of Internal Medicine is based on a detailed collection of information from each specific patient. Diagnostic hypotheses and personalized treatment plans are formulated by integrating data from a comprehensive clinical interview, the patient’s medical history, physical examination, and findings from complementary tests [1]. This meticulous process is vital to delivering high-quality care and ensuring patient safety [2]. Additionally, the complexity of patients in internal medicine sets this specialty apart from others [3]. Patients are often elderly, present with multiple comorbidities, and their health is managed with multiple chronic medications. When hospitalized, these patients require high-touch or high-intensity care resources, such as additional diagnostic tests and multiple treatments, which in turn can have significant clinical and economic consequences. In this challenging scenario, a well-designed clinical decision support system that synthesizes the large volumes of patient data collected or generated during usual care can guide decision-making [4,5,6,7,8,9]. Artificial Intelligence is a promising tool in this regard. One of its subsets, Generative AI (GAI), is especially worthy of in-depth exploration.

The European Federation of Internal Medicine (EFIM) recognizes the transformative potential of AI in advancing the field of internal medicine. This position paper from the EFIM explores how AI can be applied to achieve the goals of P6 Medicine principles in internal medicine [10]. Medicine integrates a holistic approach to patient treatment and considers a wide range of factors to provide increasingly effective preventive and medical care [6,7,8]. Together, AI adoption, especially generative artificial intelligence (GAI) and digital therapeutics (DTx), and a focus on psycho-cognitive factors for patients paves a path towards an integrated, data-driven future for healthcare Data can be collected via the Internet of Medical Things (IoMT), a network comprising connected medical devices, sensors, and software that gather, share, and analyze real-time health information [9]. Although AI is promising in medical applications, its adoption in Internal Medicine may be inconsistent and unregulated. Specific issues of concern about AI applications include clinical efficacy and usability, ethical responsibility, and legal compliance [10,11,12,13,14]. Also, there is still a lack of clear, practice-oriented guidelines for internists on how to incorporate these technologies safely, effectively, and ethically into patient care [10,11,12,13,14,15,16,17,18,19].

### 1.1. Generative AI in Internal Medicine

Artificial intelligence (AI) applications demonstrate the capability of improving clinical decision-making by enhancing clinical accuracy [11] through the detection of patterns and changes in medical data [12]. Additionally, AI facilitates personalized treatments by using longitudinal parameters and continuous symptom tracking [12]. GAI shows significant potential benefits in medical applications, however its overall effectiveness and usability in clinical practice remain to be fully validated. GAI can generate new content from existing data patterns, hence, it can be used for generating images, text or even synthetic patient data for any purpose, like medical education, clinical decision support systems in the form of chatbots or synthetic patient data for research [18]. In terms of diagnostic effectiveness, GAI can solve simulated medical cases at rates that are comparable to or even higher than clinicians [19]. In a randomized clinical trial, clinicians who were directly supported by GAI improved their overall diagnostic performance [20]. Similarly, a systematic review and meta-analysis across ten medical databases found that ChatGPT, (version GPT-4) as a specific form of GAI, achieved an accuracy of 56% in addressing medical queries, although with considerable variability depending on specialty and task [21]. Regarding communication-related applications, such as drafting discharge instructions and preparing patient messages, GAI is effective to generate texts that are high in empathy and readability, potentially reducing clinician workload [22]. GAI also shows promise in supporting clinicians in tasks like information-gathering and summarization [23], such as summarizing a patient history from the electronic health record (EHR) [24].

In specific specialties, GAI can also train on medical and multiomics databases to design highly personalized treatment plans for patients affected by comorbidities or complex conditions, such as rare genetic disorders and cardiovascular diseases. GAI can monitor biomarkers longitudinally and optimize pharmaceutical prescriptions with the improvement of patient care [25,26]. GAI in medical imaging and radiology applications can improve image quality by synthesizing clearer pathology images and training more robust diagnostic models. Promising techniques include Generative Adversarial Networks (GANs) and diffusion models, which allow the generation of high-fidelity medical image reconstructions [27]. Other examples of GAI use in medical research include clinical trial simulation, disease outbreaks modelling, and drug development assistance [28] or conducting pilot studies to evaluate the quality and accuracy of ChatGPT-generated answers to common patient questions [4,29]. In education, GAI could help medical students practice collecting a patient history with virtual patients that are AI chatbots [30]. Digital Therapies (DTx) (for example, Youper 12.04.000), which are AI software applications for disease or disorder prevention and management, are used in the clinical setting to influence patients’ health behaviors and health outcomes, similar to how drugs interact with a patient’s physiology [31]. Like a prescribable medication, DTx consists of an active ingredient (algorithm) and one or more excipients (dense dynamic interactions). DTx aims to correct dysfunctional behaviors through cognitive therapies and can pair with devices, sensors, or wearables. DTx is mostly prescription-based once rigorously evaluated for effectiveness and also collects or generates real-world data for continuing analytics that support therapy optimization [8].

However, Generative AI faces challenges when it comes to integration into existing workflows due to missed instructions and variable accuracy depending on the pathology or discipline [23]. Successful integration also relies on social or organizational factors, such as training and oversight rather than solely on the GAI application or model [32]. Importantly, GAI is known to generate hallucinations, or fabricated outputs, which could have serious health care consequences. For example, hallucinations were detected in 6% of GAI-generated discharge summaries, while 18% presented potential patient safety issues [33]. GAI models also exhibit sociodemographic biases, which arise from their training data and algorithms (also known as data bias or algorithmic bias), which raises the risk of perpetuating health system biases and inequities [34].

For these reasons, the GAI applications in medicine require constant human supervision to prevent errors, bias, and harmful consequences for patients, or a human-in-the-loop approach. GAI should be a supportive tool in patient treatment and management, rather than an autonomous agent [23]. AI literacy for clinicians who use GAI will become a necessary competency for development and training, so that they can effectively formulate queries and critically interpret outputs and recognize potential system failures, including hallucinations and bias [25]. GAI design, instruction, and implementation in a healthcare context should be adapted to the needs of individual organizations and medical departments [10]. This includes the potential for AI agents to be applied in clinical practice. Privacy and ownership of health data are also important considerations due to corporate ownership of GAI platforms, which are privately owned even if publicly available for use, and governed by regional or national laws and regulations [35]. As a result, GAI applications must strictly comply with government regulations and established frameworks to protect privacy and data ownership issues and guarantee transparency and accountability in patient care [36,37].

### 1.2. EFIM Position on AI in Internal Medicine

The European Federation of Internal Medicine (EFIM) identified an urgent need to close the gap between emerging technologies and their practical applications in clinical internal medicine settings [38]. This position paper offers professional guidance for internal medicine practitioners. We aim to:

- Provide practical and immediate recommendations for internal medicine specialists: Offer advice on how to include AI in the clinical workflow within a department or clinic, and with other medical disciplines. AI can also be employed to support the daily clinical activities and reduce administrative workload.
- Assure that human expertise and value is not overlooked and that physician well-being is supported: Core clinical skills and medical professional competencies remain a foundation on which digital literacy and AI literacy should be built.
- Reduce uncertainty regarding ethical, legal, and data privacy issues: Internists require clear frameworks to manage risks such as algorithmic bias, liability, compliance with regulations like the General Data Protection Regulation (GDPR), patient consent, and transparency.

Recommendations drawn from rigorous studies of innovative technologies allow for evidence-based applications of AI, including GAI, and DTx. This avoids misleading information, reliance on hype, unrealistic expectations, misallocation of resources, and loss of trust in scientific and medical advancements. Furthermore, robust scientific investigations, including pilot studies where appropriate and ethical, allow for application developments that account for patient safety, data privacy, data and algorithmic biases, and accountability in controlled environments before widespread adoption. They also offer pathways towards justifying investment in GAI research and implementation in healthcare systems.

This EFIM position paper describes the approach to evidence synthesis and recommendations for internal medicine specialists on how AI can be applied to achieve the goals of P6 Medicine principles in internal medicine.

## 2. Methodology

**Figure 1:**
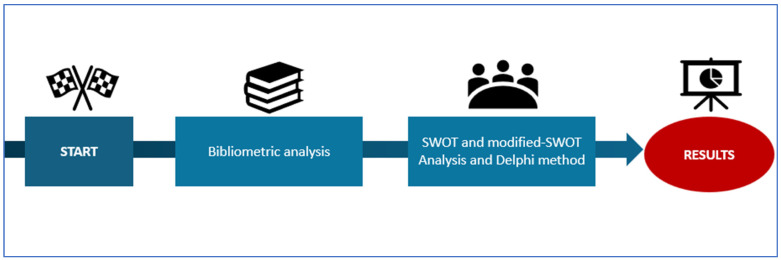
Methodology diagram.

To develop an evidence base for analysis, this position paper involved the following key approaches: a literature search and bibliometric analysis; a Delphi consensus development among a group of 30 European internal medicine AI specialists and digital health experts; and a SWOT analysis. The methodology diagram was shown in Figure 1.

### 2.1. Literature Review

Literature examining the relationship between AI and Internal Medicine was retrieved through a bibliometric analysis [39]. We iteratively refined the search approach with different keywords and analyzed the retrieved citations for relevance to the study aim. Only English language peer-reviewed literature was included. The search was performed on 2 April 2024, using Elsevier’s SCOPUS search engine. The query used was: TITLE-ABS-KEY (“artificial intelligence” OR “machine learning” OR “deep learning” OR “AI”) AND TITLE-ABS-KEY (“internal medicine” OR “clinical medicine” OR “internal diseases” OR “internal health”) AND TITLE-ABS-KEY (“cost” OR “costs” OR “expenditure” OR “financial impact” OR “economic impact” OR “expenses”) AND TITLE-ABS-KEY (“benefit” OR “benefits” OR “advantages” OR “improvements” OR “outcomes”). The definitions present on MESH (National Library of Medicine portal) were adopted: https://www.ncbi.nlm.nih.gov/mesh/ (accessed on 2 April 2024).

The bibliometric analysis was performed using the R package, version 4.4.1 bibliometrix [40]. Analysis involved network analysis and multiple correspondence analysis, a statistical methodology (a statistical multivariate method which helped to synthesize the results which allow to identify the thematic communities of keyword), to build conceptual maps that help identify the specific relationships between critical keywords in the literature [15]. To determine the semantic cores of the literature and strengthen the findings from each methodology, we have performed a hierarchical cluster analysis based on different community results obtained from the concurrent keyword networks [16]. At the same time, following this approach [16], we have validated the different clusters obtained.

### 2.2. Delphi Method

Themes identified from the literature review were further examined employing the Delphi method [41,42,43], centered to respond to the Grand Tour Question: “What role should Artificial Intelligence play in the care of Internal Medicine patients?”.

**Figure 2:**
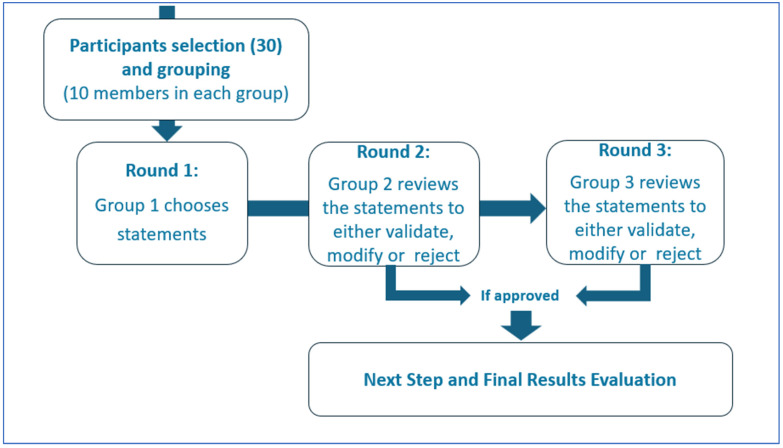
Delphi Method.

The Delphi process allows for a participatory and shared process between experts (both clinical and technical) similar to Socrates’ maieutics in order to highlight the different facets of the topic under study (Figure 2).

Thirty international AI and Internal Medicine experts participated to the Delphi panel, including members of the EFIM Telemedicine, Innovative Technologies and Digital Health Working Group and other professionals with relevant academic and clinical experience [42]. The panel was divided into three groups of ten experts. The first group selected the initial statements for investigation; the second group then reviewed the statements to either validate, reject, or modify them. The validated statements were included in the Strength, Opportunity, Weakness and Threat (SWOT) analysis, while the third group analyzed the rejected or replaced statements [44]. Each statement was scored for the perceived value and risk using a Likert scale from −10 to +10 [43]. The median scores were used to determine consensus and plotted x (risk) and y (value) axes to identify strengths (−x/+y), weaknesses (−x/−y), threats (+x/−y), and opportunities (+x/+y) [18]. Panel discussions were carried out in person or remotely during video calls (via Zoom or Google Meet). Participants scored the statements via a Google Form; they could not view other participants’ individual responses. One author (FR) had access to the results at the end of the third round, and also conducted the statistical survey. As consensus was reached between the first and second group of experts, input from the third group of Delphi’s panel was not necessary. Open Science Framework, however, does not allow Italy to be selected as a repository for the data.

## 3. Research Outcomes

**Figure 3:**
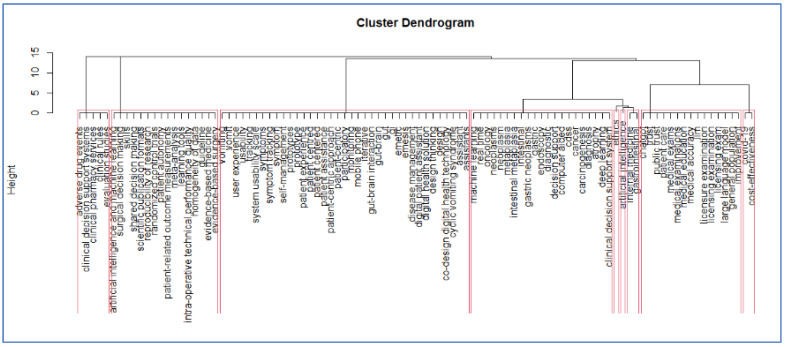
The final dendrogram of the bibliometric analysis. Each red rectangle shows a different cluster of keywords reflecting the current debate in the field.

The methodology described in the paper is based on a comprehensive review of relevant literature and the identification of key themes through hierarchical clustering on the application of AI in medicine. The resulting dendrogram (Figure 3) shows the different fields of application and their integration in clinical practice.

Network analysis of the co-occurring keywords and distinct phases, such as multiple correspondence analysis, a statistical multivariate method which helped to synthesize the results which allow to identify the thematic communities’ keywords. The dendrogram summarizes the results of the bibliometric analysis, showing the different semantic cores of literature as observed in Figure 1. Each red rectangle shows a different group of keywords reflecting the current debate in the field. Because the dendrogram is a tree-like diagram created by hierarchical grouping, it shows how the data points are grouped in sequence; each “leaf” of the diagram is a datapoint linked to other datapoints through branches of larger keyword groups. In our case at the bottom of the diagram, each data point is represented as a single “leaf”. As it is possible to move up words, the branches connect to show which elements or groups of key words come together to form larger groups of keywords. Figure 1 shows where there may be relevant debates in this field of AI applications in Internal medicine. In order to interpret the dendrogram, two different keywords which are in the same branch of the dendrogram are keywords which appear closely in the scientific literature, for instance these topics are discussed jointly in articles. More generally it is possible to say that keywords which appear in the same cluster or groups are related similar debates in literature (they appear closely in different publications).

The bibliometric analysis emphasizes the relevance of AI in Internal Medicine, particularly its role in earlier disease detection, which can reduce healthcare costs and improve patient treatments [45]. In addition, AI can improve clinical workflows and reduce the risk of medical errors [46]. AI also facilitates personalized medicine with tailored treatment strategies [5]. However, AI can result in relevant costs, which include not only its initial integration into medical practice, but also the ongoing need for updates and maintenance.

**Figure 4:**
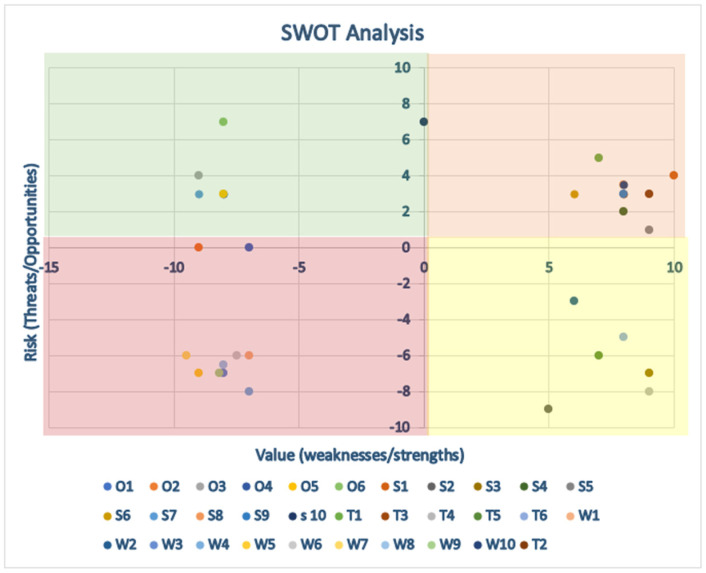
Represents the quantitative scores from the SWOT analysis. Green area: positive area. Yellow area: items can be improved or more attention is required to apply them. Red area: dangerous area.

The Delphi results were summarized in a SWOT Analysis, as shown in Table 1 and Figure 4. Substantial comments were included in the SWOT Analysis, while textual clarifications were addressed within the panel.

**Figure 5:**
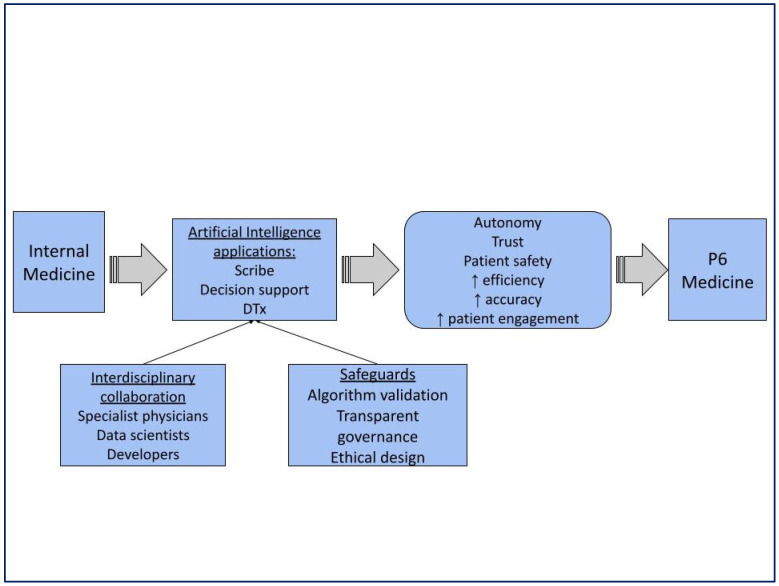
Summarizes the main outcomes of the Delphi consensus and relates them to P6 medicine. The arrows show how the use of AI contributes to the realization of the P6 Medicine increasing autonomy, trust, patient safety, efficiency, accuracy and patient engagement.

The in-depth discussion that took place within the groups allowed us to highlight the main shared conclusions that the possibility of increasing the capacity to analyze data significantly improves diagnostic capacity, the development of therapies and patient tracking (Figure 5).

The consensus panel unanimously acknowledged AI in Internal Medicine as an opportunity. Fewer opportunities for improvement emerged and showed polarized views, as represented in the graph by a clustering of points toward the outer vertices, which is typical of disruptive technologies.

### 3.1. Consensus Recommendations on Generative AI in Internal Medicine

In summary, GAI can be trained to generate real-time content, such as text, images, or even synthetic patient data, based on patterns from existing data. In internal medicine, GAI offers promising applications, including the following:

- Research and Drug Discovery: GAI can generate synthetic patient data for clinical trial simulations and accelerate drug development. For instance, it can assist in drug development by identifying new therapeutic compounds or simulating their effects.
- Personalized Medicine: GAI can generate synthetic data to train AI models for more accurate personalized medicine applications, leading to tailored treatment plans.
- Population Health Management: GAI has the potential to model disease outbreaks and develop more effective public health interventions. By simulating different scenarios, internists and public health officials can proactively design strategies to minimize the impact of infectious diseases and chronic conditions in populations.
- Improving Clinical Documentation: GAI provides new methods for introducing information into clinical records, such as voice recognition or predictive writing, which can significantly improve the documentation process and reduce the associated workload and professional burnout [7].
- Clinical Decision Support and Patient Education: GAI can be used for clinical decision support systems in the form of chatbots or for medical education, such as generating images, text, or synthetic patient data for history taking in virtual patients. Pilot studies have evaluated the quality and accuracy of ChatGPT-generated answers to common patient questions [8].

Generative AI offers a pathway for internal medicine specialists to advance P6 Medicine goals and improve overall efficiency.

### 3.2. Recommended Integration of AI into Clinical Processes

Successfully integrating AI into clinical workflows requires careful consideration and a detailed analysis of existing processes by internists and healthcare organizations. The aim is to identify areas where AI solutions can improve the quality of care and reduce documentation burden. Examples of practical applications include:

- AI-powered Scribes: These tools automate documentation tasks, such as compiling clinical information registries or generating clinical notes, potentially reducing administrative burden and freeing up attention and time for the internal medicine specialist to focus on patient interaction [45].
- Clinical Decision Support Systems (CDSS): AI-powered CDSS can provide real-time guidance to internists at the point of care, improving diagnostic accuracy and treatment efficiency by analyzing vast datasets and offering evidence-based recommendations [46].
- Digital Therapies (DTx): Examples include DTx for preventing hospital visits, improving patient self-management, and providing remote therapy. DTx may facilitate increasing patient participation and empowerment, continuous monitoring, feedback, and behavioral support in medical treatments. Physicians can serve patients as a guide, interpreting data, personalizing treatments, and coordinating effective integration of digital treatments into health pathways, especially for patients with lower digital skills [24,47]. From an economic perspective, DTx could lead to cost-saving opportunities through increasing treatment adherence, reducing acute exacerbations of chronic conditions, offering scalable interventions, and improving healthcare service efficiencies [47].

Key areas for AI integration in internal medicine include tools such as medical scribes, clinical decision support systems (CDSS), digital therapeutics, and treatment adherence, offering clear opportunities for improving efficiency, accuracy, and patient engagement. The strengths highlighted in the SWOT analysis suggest significant potential for improving both clinical workflows and patient outcomes. At the same time, the analysis emphasizes that these benefits cannot be realized in isolation: careful algorithm validation, seamless workflow integration, and transparent governance are essential to ensure safety and trust. Ethical issues such as equity and patient autonomy must be considered, recognizing that poorly designed or implemented AI tools risk reinforcing disparities or undermining patient-centered care. Overall, the analysis positions AI as a transformative force in internal medicine, but its success depends on responsible, deliberate, and ethically grounded implementation. Applied appropriately in this way, internal medicine specialists may be allowed more effort and time to attend to complex patient care.

To advance the field, GAI applications can be evaluated in various ways in both pilot and large-scale settings. Example GAI evaluations could focus on administrative efficiencies (e.g., reduced documentation time or prescribing and ordering time), factual accuracy or errors (e.g., in medical history summaries), usability and acceptability by healthcare professionals and patients, and the introduction or perpetuation of systemic biases and inequity. DTx, as a specific AI application, also deserves evaluation due to heterogeneity of endpoints, which limits generalizability. Use of primary endpoints would improve the robustness of DTx evaluation. For example, primary endpoints are the Insomnia Severity Index for insomnia assessment, and HbA1c changes between two clinically meaningful time points for diabetes monitoring. Ultimately, randomized, parallel-group trials where ethically possible would be a gold standard for evaluating all possible AI applications and DTx. Therefore, future studies should concentrate on primary endpoint examples.

### 3.3. Legal, Regulatory, and Ethical Considerations

Legal, regulatory, and ethical considerations are vital in the potential use of AI, particularly GAI, in healthcare [48]. Many of the challenges internal medicine specialists face are universal for all clinicians. First, AI tools are required to comply with strict data privacy regulations, such as the General Data Protection Regulation (GDPR) in the EU [49]. In the current environment, this is an evergreen challenge to assure protection of sensitive patient information [50]. However, discrepancies in the interpretation of regulations across different regions and countries results in variability in compliance, and ongoing vigilance and collaboration may allow for pathways towards uniform regulatory interpretations. Additionally, robust cybersecurity measures involving AI will also need to evolve in contemporary times where data breaches, hacking attempts, and new threats also appear. This could include clinicians developing competencies and awareness of new protocols, and organizations can engage in routine security audits [46].

The European Commission has established a comprehensive framework to promote the safe, ethical, and reliable use of AI in healthcare [37,38]. This framework sets out key principles that are crucial considerations for AI applications in internal medicine:

- Transparency: AI models should be designed to allow clinicians to understand decision-making processes.
- Fairness: AI must operate without bias and should support equal access to care.
- Accountability: Clear responsibility must be assigned for AI development and implementation.

These principles are embedded in the EU Artificial Intelligence Act (AI Act) in March 2024, and sets out detailed requirements for high-risk AI applications, including those in healthcare. However, some regulatory gaps remain, particularly concerning DTx, which is by design a software-based solution. DTx are categorized as Medical Device Software (MDSW) under the EU Medical Devices Regulation 2017/745 (MDR). Despite this, the regulation does not address them. Uncertainties about DTx classification and market authorization could lead to misclassification as low-risk, even if DTx has a high therapeutic impact. To address these gaps, frameworks like the “Software as a Medical Device: Possible Framework for Risk Categorization and Corresponding Considerations,” developed by the International Medical Devices Regulators Forum (IMDRF), can be beneficial [51].

Second, liability for errors occurring as a result of AI applications remains uncertain: whether the technology developer, healthcare organization, or individual clinician is liable for errors is yet to be determined. Whether AI can be liable is also unclear, given that there are already some industries where AI, specifically GAI, cannot be accountable for its outputs.

Third, explainable AI is founded on transparency for the user to sufficiently understand how the model works in generating an output, such as a decision support recommendation. This includes aspects of bias and equity, which may involve explainability also of the source data for developing and training AI.

Finally, AI is seen best positioned as augmenting human expertise, rather than replacing it. The human-in-the-loop model would be relevant to achieve the most gains from AI applications. This also may best support productive therapeutic patient-physician relationships in an increasingly automated environment.

**Figure 6:**
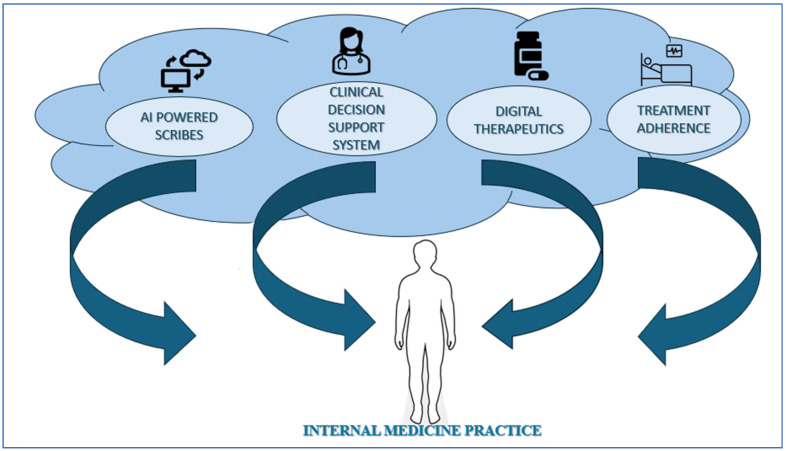
Impact of AI on Internal Medicine practice.

To summarize, AI applications in Internal Medicine offer significant opportunities to improve both efficiency and quality of care and reduce administrative burdens. The EFIM sees opportunities and challenges in AI applications advancing P6 Medicine principles in the field, evolving and improving care effectiveness. Figure 6 highlights the impact of AI on Internal Medicine practice.

The EFIM provides an official statement and key recommendations for the implementation of AI in internal medicine (Table 2). These recommendations are designed to guide internal medicine specialists and their organizations in using AI responsibly and effectively to improve patient care.

Several key points were identified by the panel as fundamental elements for AI use in Internal Medicine:

- Increasing medical data poses a significant administrative challenge.
- Organizing information is essential for digitizing clinical workflows, such as clinical decision support systems.
- A comprehensive and punctual collection of clinical data allows to design AI-driven decision support tools that integrate into internal medicine practices.
- Ethical and responsible data collection is essential to ensure patient privacy and consent to maintain patient safety and trust [54].

## 4. Conclusions

AI applications, including GAI and DTx, have the potential to transform internal medicine by streamlining workflows, enhancing patient care, and generating valuable data. These technologies offer a path towards more efficient, personalized, and human-centered healthcare. However, successful integration of such technologies in the healthcare domain hinges on interdisciplinary collaboration. Care teams should involve new professional roles, such as clinical data scientists and bioengineers. Additional fundamental requirements are adherence to evolving EU regulations and focus on ethical considerations like transparency and fairness. Patients and their caregivers must remain at the care center delivery. As a call to action, the EFIM recommends that internal medicine specialists, along with their organizations, professional societies, and communities, take a leading role in shaping the education, design, implementation, evaluation, and regulation of AI in clinical settings.

## Figures and Tables

**Table 1:** SWOT Analysis: AI used in Internal Medicine.

**Strengths**	**Weaknesses**
S1. Extensive data analysis: AI can recognize patterns in extensive clinical datasets and improve diagnostic accuracy. S2. Early diagnosis: By detecting subtle signs in medical images (X-rays, CT scans, MRI), AI can identify early signs of medical symptoms. S3. Development of new drugs: AI accelerates drug development, cutting down traditional research time and costs. S4. Personalized care: AI facilitates personalized treatment plans based on genetic and individual profiles. S5. Improved Accessibility: AI improves access to medical services in remote areas. S6. Lower healthcare costs: AI can decrease hospital admissions and specialist visits. S7. Increased patient engagement: Digital therapeutics (DTx) encourage an active role in managing personal health. S8. Continuous access to care: AI apps enable patients to receive support 24/7. S9. Real-time data monitoring: DTx enable personalized treatment adjustments. S10. Improved treatment tracking: AI allows physicians to monitor patient adherence and progress more accurately.	W1. Data privacy and security: It is critical to protect patient information and the security of digital platforms. W2. Limited Accessibility: Not all patients have access to or can effectively employ digital tools. W3. Regulation: A clear and shared legal framework for AI is still evolving. W4. Data dependency: AI efficacy relies on data quantity and quality for training. W5. Biased data: If AI is trained on biased data, the outcomes can be inaccurate and discriminatory. W6. High costs: Developing and implementing AI-based solutions requires significant investments. W7. Regulatory fragmentation in the EU: Substantial differences in regulatory interpretation exist across EU Member States. W8. Lack of standardized reimbursement for DTx: most countries have no defined reimbursement process for DTx that may prevent DTx adoption. W9. Insufficient funds for DTx: lack of adequate financial support for DTx.
**Opportunities**	**Threats**
O1. Decreased healthcare burden: DTx may prevent hospital visits, improve patient self-management, and provide remote therapy. O2. Advancements in Precision Medicine: Precision Medicine can rapidly evolve as AI fosters highly personalized treatments. O3. Innovative Care Models: new care models can be introduced, such as telemedicine and preventive medicine. O4. Improved human–machine cooperation: AI can function as a valuable assistant to healthcare professionals. O5. Research and Development: AI accelerates discovery of new therapies and the employment of Digital Twins technology for simulations.	T1. Regulatory uncertainty for software changes: discrepancies regarding software updates and how they influence market authorization. T2. Security and privacy risks: accurate security and privacy measures to protect patient data are still to be developed. T3. Effectiveness comparison: the effectiveness of digital therapies compared to traditional drugs is still unclear. T4. Ethical considerations: complex ethical issues arise in AI application, such as the liability in case of errors and the potential dehumanization in patient care. T5. Ethical considerations: The application of AI in healthcare raises complex ethical issues, such as the liability in case of errors and the potential dehumanization in patient care. T6. Resistance to change: The adoption of AI may face opposition from healthcare professionals and patients. T7: Technological dependence: Excessive reliance on AI-based technologies can expose healthcare systems to service disruption risks.

**Table 2:** The EFIM Official statement proposed for the implementation of AI in Internal Medicine.

**Official Statement by the European Federation of Internal Medicine on the Implementation of Artificial Intelligence**
The European Federation of Internal Medicine (EFIM) acknowledges the pressing progress of AI and its potential to disrupt medical practice. These recommendations are designed to guide internists in using AI responsibly and effectively and improve patient care.
**Section 1: General Recommendations for Using AI in Internal Medicine** 1. Relevance of Core Clinical Skills: AI should augment core competencies, rather than substitute them. Core competencies in internal medicine are accurate information gathering through patient interviews, physical examinations, and medical history review. 2. Data Processing and Conversion: Internal medicine specialists can advocate for intelligent, supportive information systems. Increasing volumes of health data poses a significant administrative challenge. AI may be able to efficiently organize information, which is essential for digitizing clinical workflows [52]. Data architectures, AI, and health information systems capable of transforming data and information bidirectionally would best support clinical and shared decision-making. 3. Streamlining Workflow and Reducing Burnout: AI applications serve to augment clinical practice, for example, by optimizing workflow and automating routine or repetitive tasks. Internal medicine specialists in turn can then increase focus on patients and healthcare organizations can reduce burnout related to administrative burdens. 4. Interdisciplinary Cooperation: Internal medicine specialists work within a collaborative team, including but not limited to healthcare professionals, data scientists, AI application developers, and patients. Interdisciplinary teamwork ensures appropriate participation of key individuals in optimal AI applications in internal medicine. 5. Data privacy and protection: Education on security protocols and regular security audits facilitate regulatory compliance, which is mandatory. Standard data privacy and security regulations apply to ensure secure AI implementation and minimize exposures [53]. Ethical and responsible data collection is essential to ensure patient privacy and consent to maintain patient safety and trust [54]. Internal medicine specialists or their healthcare organizations must ensure compliance throughout the process of AI development, use, and evaluation. 6. Ethical Concerns and Human Expertise: Human-in-the-loop approaches for AI applications should involve internal medicine specialists. Human expertise and supervision can help support identification of biases [55] and provide professional interpretation of AI outputs. 7. Liability I and AI: Liability for errors resulting from AI use remains uncertain. This is imperative across all medical specialties, and internal medicine specialists can engage in advocacy to ensure appropriate representation of this cognitive speciality in during policymaking. 8. Digital Skills and Continuous Learning: Internal medicine specialists would benefit from continuous learning and development of digital skills, including AI literacy, as technologies evolve, even if the extent and content of such a skillset is still a matter of debate [56]. Also, as AI applications are constantly evolving, internists are encouraged to stay informed on the latest advancements in AI in Internal Medicine [57].
**Section 2: Specific Recommendations for using Generative AI (GAI) in Internal Medicine** 1. Data Augmentation and Personalization: Generative AI (GAI) can enhance patient data collection to help personalized medicine strategies. This data could be applied to feed Big Data models for more precise diagnoses and tailored treatment plans. Additionally, GAI can create anonymized synthetic data sets for research, accelerating drug discovery and other healthcare innovations. 2. Population Health Management: GAI has the potential to model disease outbreaks and develop more effective public health interventions. By simulating different scenarios, internists and public health officials can proactively design strategies to minimize the impact of infectious diseases and chronic conditions in populations. 3. Transparency and Patient Consent: It is crucial for healthcare professionals to communicate clearly with patients about the role of Large Language Models (LLM) in the clinical setting. Internists should explain both the potential benefits and risks associated with GAI application and obtain informed consent from patients before implementing GAI-based tools in their care. 4. Specialization and Expertise: As GAI applications in healthcare evolve, it is advisable for several internists to specialize in areas where GAI can have a significant impact, such as precision medicine or chronic disease management.
**EFIM’s Call to Action** AI applications in Internal Medicine offer significant opportunities to improve both efficiency and quality of care and reduce administrative burdens. EFIM recommends that specialist physicians in internal medicine, along with their organizations, professional societies, and communities, take a leading role in shaping the education, design, implementation, evaluation, and regulation of AI in clinical settings.

## Data Availability

The data will be provided by the corresponding author upon request.
